# Elevated novel inflammatory markers in heart failure patients are associated with increased risk of adverse outcomes within one year: insights from a longitudinal study

**DOI:** 10.3389/fcvm.2025.1683273

**Published:** 2025-09-25

**Authors:** Zeng Zhao, Chunmei Yuan, Ting Lan, Feng Liu, Guobin Liu, Yuping He, Jing Li, Xingjun Liu

**Affiliations:** ^1^ Department of Cardiology, Jintang County First People’s Hospital, Chengdu, China; ^2^ Department of Cardiology, Sichuan Science City Hospital, Mianyang, China

**Keywords:** heartfailure, inflammatoryMarkers, re-hospitalization, death, cardiovascular diseases

## Abstract

**Background:**

Inflammation has been widely recognized as a key contributor to the pathogenesis of numerous diseases, including cardiovascular disorders. This study aims to investigate the associations between different novel inflammatory markers and adverse outcomes within one year in patients with HF, and to identify the most effective predictor.

**Methods:**

Three inflammatory markers—Systemic Inflammatory Response Index (SIRI), Systemic Immune-Inflammatory Index (SII), and Neutrophil-to-Lymphocyte Ratio (NLR)—were evaluated. Cox regression analysis was performed to examine their associations with adverse outcomes within one year. Kaplan–Meier (KM) survival analysis was used to estimate the cumulative risk of adverse events. Additionally, receiver operating characteristic (ROC) curves, time-dependent ROC curves, and C statistics were applied to compare the predictive performance of these markers.

**Results:**

All three inflammatory markers were significantly associated with adverse one-year outcomes in HF patients. For each one standard deviation increase in SIRI, SII, and NLR, the risk of re-hospitalization increased by 45.5%, 54.9%, and 63.7%, respectively, while the risk of death increased by 63.8%, 70.1%, and 92.9%, respectively. ROC analysis indicated that SIRI had superior predictive performance compared with SII and NLR, and time-dependent ROC results further confirmed its stronger prognostic value.

**Conclusion:**

This study demonstrate that several novel inflammatory markers are strongly associated with adverse one-year outcomes in HF patients. Comparative analysis revealed that SIRI provides the most robust predictive performance, highlighting its potential as a valuable clinical tool for monitoring and risk stratification in HF management.

## 1 Introduction

Heart Failure (HF) is a condition characterized by impaired cardiac function, preventing the heart from effectively pumping blood to meet the body's needs. This dysfunction leads not only to multi-organ failure but also significantly diminishes patients' quality of life (1, 2). As epidemiological data continues to evolve, the prevalence of HF has steadily increased, particularly among the elderly, making it a growing public health concern worldwide (3). More importantly, HF patients not only face a high mortality risk but also experience frequent hospitalizations and long-term health complications, which place a substantial strain on healthcare systems (4, 5). Consequently, early identification of high-risk individuals, improving long-term outcomes, and reducing readmission and mortality rates have become key priorities in current clinical research.

In the pathogenesis of HF, inflammation plays a crucial role. Inflammation is a protective response of the body to injury or infection, and it is not only involved in the initiation and progression of HF but is also strongly associated with the poor clinical prognosis of various diseases (6–8). This is especially true in cardiovascular diseases (CVD), where inflammation contributes to the development and maintenance of hypertension, atherosclerosis, and the rupture of arterial plaques (9–11). These findings suggest that controlling inflammation could offer a new strategy for preventing CVDs and their adverse outcomes.

Traditional inflammation assessment primarily relies on single indicators such as white blood cell count, C-reactive protein (CRP), and procalcitonin. However, these markers often fail to provide a comprehensive view of the body's inflammatory state. In contrast, newer inflammatory indices based on blood cell counts have gained attention due to their low cost and ease of use (12–15). For example, ratios such as the monocyte-to-lymphocyte ratio (MLR), platelet-to-lymphocyte ratio (PLR), and neutrophil-to-lymphocyte ratio (NLR) have demonstrated independent predictive value in assessing the prognosis of various diseases (14, 16, 17). More comprehensive markers, such as the systemic immune-inflammation index (SII) and systemic inflammatory response index (SIRI), offer a more accurate assessment of systemic inflammation by integrating data from multiple immune pathways. Studies have shown that SIRI not only predicts the course of inflammatory diseases but also shows promise in predicting the progression of cardiovascular diseases, metabolic disorders, and stroke (9, 12, 14). Notably, PLR is closely linked to the onset of hypertension (17), while elevated SII and SIRI levels are significantly associated with an increased risk of stroke, osteoporosis, and kidney disease progression (14, 18). Additionally, these indices have been shown to correlate with the occurrence of fatty liver disease and liver fibrosis (9). These innovative inflammatory markers, derived from routine blood cell counts, reflect the body's multi-pathway inflammatory and immune status, offering new avenues for early disease detection and prognosis assessment.

Building on this research background, the aim of this study is to systematically investigate the association between novel inflammatory markers and adverse outcomes in HF patients within one year. The study will focus on identifying the most predictive inflammatory indicators, thereby providing a scientific basis for early clinical monitoring and intervention, and optimizing treatment strategies for HF patients.

## 2 Material and methods

### 2.1 Screening of the study population

**Figure 1:**
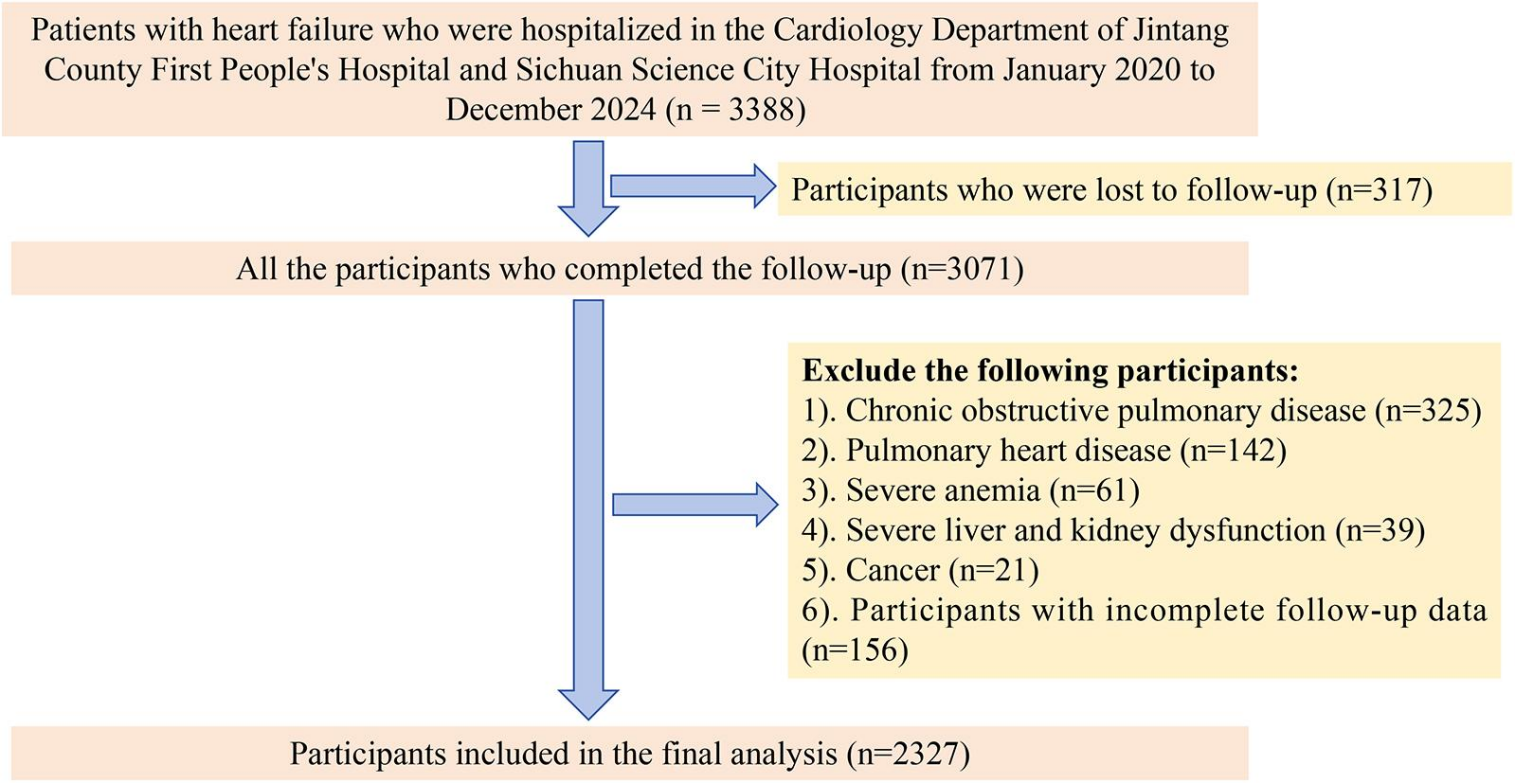
Flowchart for selection of study populations.

This study adopted a dual-center longitudinal cohort design and included patients diagnosed with HF who were hospitalized at the First People's Hospital of Jintang County and at Sichuan Province Science City Hospital between January 2020 and December 2024. The inclusion criteria for the study were as follows: (1) reduced ejection fraction HF, defined as a left ventricular ejection fraction (LVEF) < 40%; (2) a heart function classification of III to IV, representing varying degrees of HF severity; and (3) completion of the follow-up. To account for the potential impact of other underlying conditions on the outcomes, we excluded patients with chronic obstructive pulmonary disease, pulmonary heart disease, severe anemia, severe liver or kidney dysfunction, or cancer. Additionally, patients with incomplete follow-up data were also excluded. After applying these inclusion and exclusion criteria, a total of 2,327 patients were eligible for the study (Figure 1).

This study was conducted in accordance with the principles of the Helsinki Declaration and received approval from the Ethics Committee of the First People's Hospital of Jintang County (No. 20190913001). All participants provided written informed consent.

### 2.2 Data collection and definitions

The participants' basic information, physical examination results, medical history, medication usage, and laboratory test data were collected from the hospital's electronic medical records and telephone follow-up records. Basic information, such as height, weight, blood pressure, and body mass index (BMI), is detailed in the Supplementary Materials. Laboratory test data included blood routine tests, alanine aminotransferase (ALT), aspartate aminotransferase (AST), brain natriuretic peptide (BNP), total cholesterol (TC), triglycerides (TG), fasting blood glucose (FBG), high-density lipoprotein cholesterol (HDL-C), low-density lipoprotein cholesterol (LDL-C), and albumin, all measured using an automatic biochemical analyzer. LVEF was assessed by experienced ultrasound technicians following a standardized protocol. The definitions of diseases such as coronary heart disease (CHD), diabetes mellitus (DM), and dyslipidemia were based on current diagnostic criteria, which are described in more detail in the Supplementary Materials.

### 2.3 Calculation of new inflammatory markers

Based on the results of relevant blood routine tests, new inflammatory markers are calculated. The specific calculation method is as follows:

$$\begin{array}{rl} & \mathrm{SIRI}=\mathrm{Neutrophil}\;\mathrm{count}\times \mathrm{Monocyte}\;\mathrm{count}/\mathrm{Lymphocyte}\;\mathrm{count}\\ & \mathrm{SII}=\mathrm{Platelet}\;\mathrm{count}\times \mathrm{Neutrophil}\;\mathrm{count}/\mathrm{Lymphocyte}\;\mathrm{count}\\ & \mathrm{NLR}=\mathrm{Neutrophil}\;\mathrm{count}/\mathrm{Lymphocyte}\;\mathrm{count} \end{array}$$

### 2.4 Study outcome

The primary outcome measure of this study is the occurrence of adverse events within one year during the follow-up period. These events include re-hospitalization and death within one year.

The clinical outcomes of the patients will be monitored continuously for 12 months after discharge through telephone follow-ups and medical records. In cases where multiple adverse events occur, the time of the first occurrence will be considered the endpoint to ensure data accuracy and consistency.

### 2.5 Statistical analysis

Participants were divided into three groups (T1, T2, and T3) based on the tertiles of various novel inflammatory markers. Univariate and multivariate Cox regression analyses were performed, and trend tests were conducted to evaluate the relationship between these markers and adverse outcomes within one year for patients with HF. Additionally, Kaplan–Meier (KM) curves were used to assess the risk of adverse outcomes among the different groups based on the tertiles of inflammatory markers, with log-rank tests performed for comparison. To further investigate the dose-response relationship between inflammatory marker levels and adverse outcomes, the restricted cubic spline (RCS) model was applied. To compare the predictive efficacy of different inflammatory indicators, we conducted a series of analyses, including receiver operating characteristic (ROC) curve analysis, time-dependent ROC analysis, and C statistics. These analyses were used to comprehensively evaluate the predictive performance of each marker for adverse outcomes.

All statistical analyses were performed using R software version 4.2.2, with a two-sided *P* value of less than 0.05 considered statistically significant.

## 3 Results

### 3.1 Baseline characteristics of the study population

A total of 2,327 patients with HF were included in this study. Based on whether they were readmitted within one year, the patients were divided into two groups, and their baseline characteristics were compared. The results are shown in Table 1.

**Table 1:** Baseline characteristics of the study population.

Characteristic	Totel	Non-re-hospitalization	Re-hospitalization	*P* value
*N*	2,327	1,503	824	
Age (years)	58.44 ± 8.28	58.51 ± 8.26	58.31 ± 8.32	0.589
Sex (%)				<0.001
Female	1,719 (73.87%)	1,053 (70.06%)	666 (80.83%)	
Male	608 (26.13%)	450 (29.94%)	158 (19.17%)	
BMI (kg/m^2^)	25.96 ± 3.97	25.60 ± 3.96	26.61 ± 3.90	<0.001
SBP (mmHg)	144.70 ± 18.65	144.38 ± 18.27	145.30 ± 19.32	0.256
DBP (mmHg)	88.27 ± 13.86	87.70 ± 13.26	89.31 ± 14.85	0.007
Smoking (%)	351 (15.08%)	189 (12.57%)	162 (19.66%)	<0.001
Drinking (%)	280 (12.03%)	129 (8.58%)	151 (18.33%)	0.002
Laboratory tests				
ALT (U/L)	18.00 (13.00–29.60)	17.00 (12.00–27.00)	21.37 (14.76–32.93)	<0.001
AST (U/L)	19.00 (15.00–24.16)	18.00 (15.00–24.00)	20.00 (16.00–26.00)	<0.001
Albumin (g/L)	40.60 ± 3.54	40.26 ± 3.48	41.23 ± 3.57	<0.001
TC (mmol/L)	4.12 ± 0.94	4.09 ± 0.91	4.19 ± 0.99	0.013
TG (mmol/L)	1.20 ± 0.98	1.09 ± 0.88	1.41 ± 1.13	<0.001
HDL-C (mg/dl)	1.13 ± 0.28	1.15 ± 0.28	1.09 ± 0.28	<0.001
LDL-C (mg/dl)	2.66 ± 0.78	2.66 ± 0.79	2.65 ± 0.76	0.731
FPG (mmol/L) (mmol/L)	4.69 ± 0.87	4.72 ± 0.92	4.65 ± 0.77	0.091
BNP (pg/ml)	366.30 (182.08–850.80)	366.30 (182.08–850.80)	366.30 (182.08–850.80)	<0.001
	337.20 (171.80–723.10)	337.20 (171.80–723.10)	337.20 (171.80–723.10)	
	457.38 (226.37–1,003.48)	457.38 (226.37–1,003.48)	457.38 (226.37–1,003.48)	
SIRI	0.84 (0.57–1.23)	0.74 (0.52–1.00)	1.30 (0.75–1.88)	<0.001
SII	482.23 (344.22–673.71)	436.71 (320.34–581.88)	628.61 (418.13–891.29)	<0.001
NLR	2.00 (1.55–2.65)	1.84 (1.47–2.32)	2.49 (1.82–3.49)	<0.001
Medical history				
DM (%)	180 (7.74%)	121 (8.05%)	59 (7.16%)	0.442
Dyslipidemia (%)	1,202 (51.65%)	652 (43.38%)	550 (66.75%)	<0.001
CHD (%)	765 (32.87%)	457 (30.41%)	308 (37.38%)	<0.001
Medications				
Statins (%)	1,573 (67.60%)	915 (60.88%)	658 (79.85%)	<0.001
Antiplatelet medication (%)	765 (32.87%)	457 (30.41%)	308 (37.38%)	<0.001
Diuretics (%)	2,290 (98.41%)	1,494 (99.40%)	796 (96.60%)	<0.001
Beta-blockers (%)	1,902 (81.74%)	1,242 (82.63%)	660 (80.10%)	0.130
Calcium channel blockers (%)	1,217 (52.30%)	670 (44.58%)	547 (66.38%)	<0.001
ACEIs/ARBs (%)	2,034 (87.41%)	1,306 (86.89%)	728 (88.35%)	0.311

Data are presented as mean ± standard deviation, median (interquartile range), or as numbers, and percentages.

BMI, body mass index; SBP, systolic blood pressure; DBP, diastolic blood pressure; ALT, alanine transaminase; AST, aspartate transaminase; HDL-C, high-density lipoprotein cholesterol; LDL-C, low-density lipoprotein cholesterol; TC, total cholesterol; TG, triglyceride; FPG, fasting plasma glucose; SIRI, systemic inflammation response index; SII, systemic immune-inflammation index; NLR, neutrophil-to-lymphocyte ratio; DM, diabetes mellitus; CHD, coronary heart disease; ACEIs, angiotensin-converting enzyme inhibitors; ARBs, angiotensin receptor blockers.

The average age of the study population was 58.44 ± 8.28 years, with 73.87% of participants being female, a relatively high proportion. When comparing the two groups, patients who were readmitted had significantly higher BMI and DBP, and were more likely to be current smokers and drinkers. Biochemically, patients who were readmitted had higher levels of ALT, AST, albumin, TC, and BNP, while their HDL-C was lower. In terms of medical history, the prevalence of CHD and dyslipidemia was significantly higher in the readmitted group. Additionally, these patients were more likely to be on medications such as lipid-lowering drugs, antiplatelet agents, diuretics, and other drugs aimed at improving ventricular remodeling. Notably, various new inflammatory markers were also significantly elevated in the readmitted group. Apart from these factors, no significant differences were found in other variables.

### 3.2 Incidence of adverse outcomes across different groups

**Figure 2:**
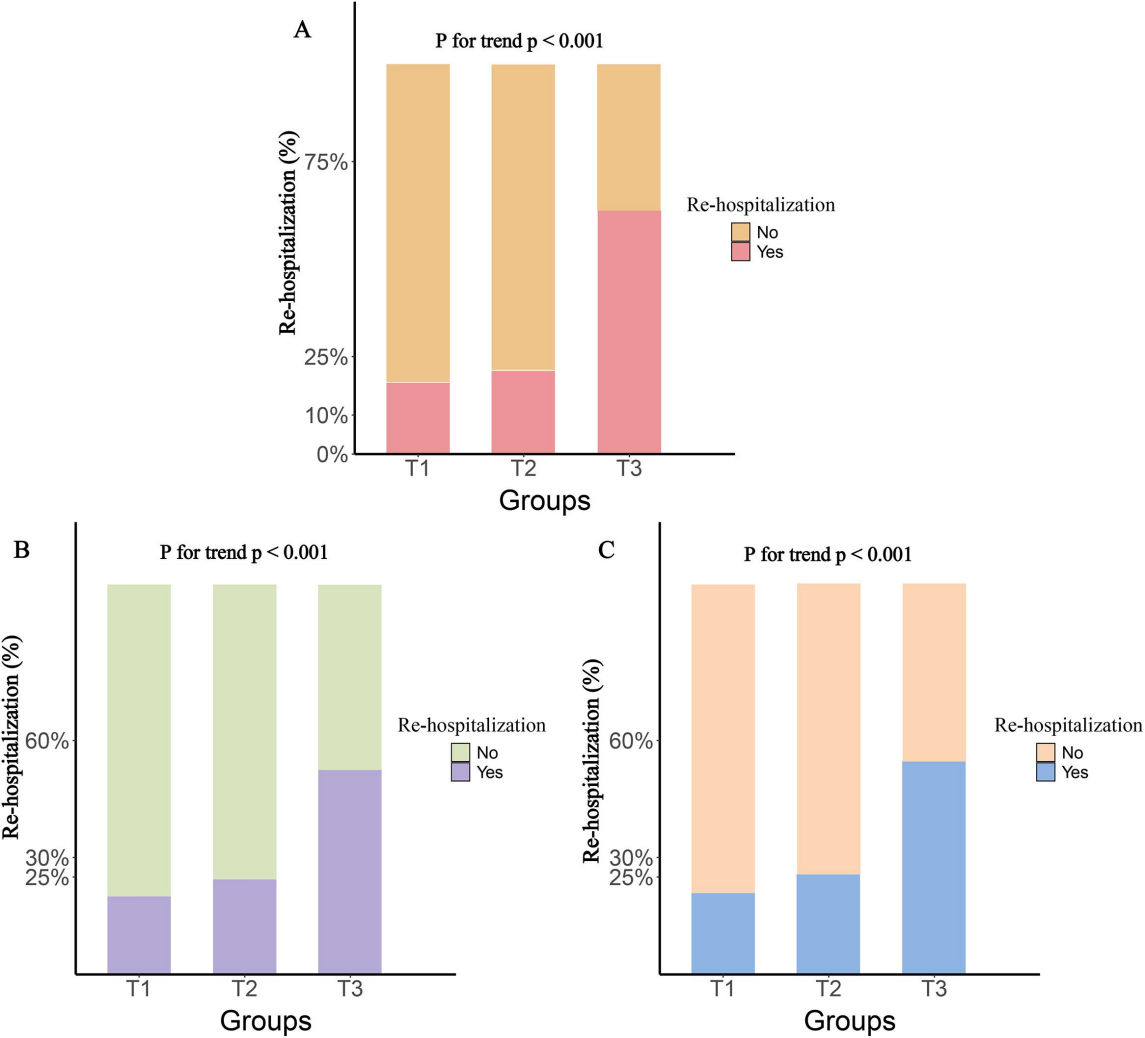
One-year rehospitalization rates by tertile groups of different inflammatory markers. **(A)**, SIRI; **(B)**, SII; **(C)**, NLR.

**Figure 3:**
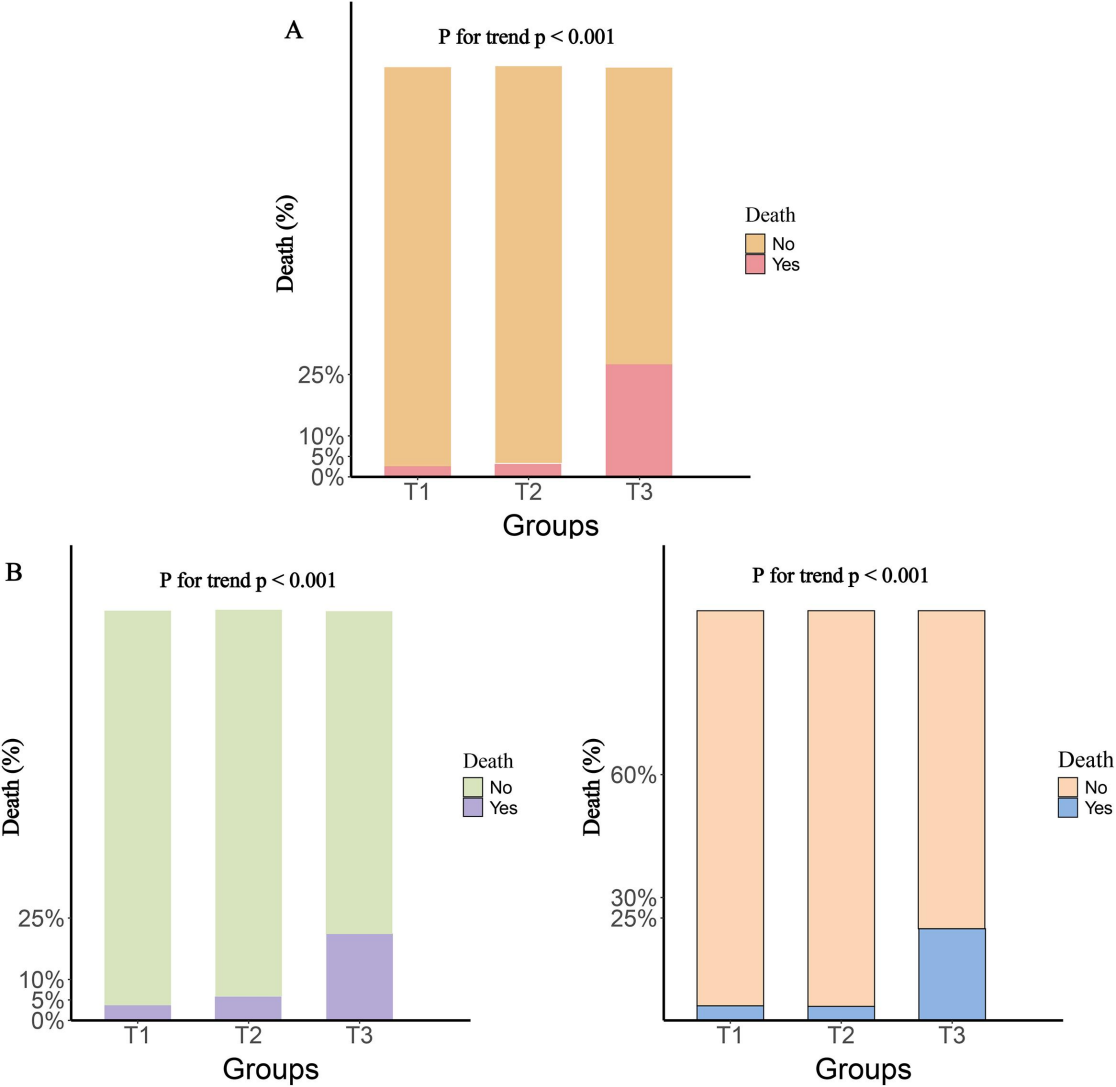
One-Year death rates by tertile groups of different inflammatory markers. **(A)**, SIRI; **(B)**, SII; **(C)**, NLR.

The participants were divided into three groups based on the tertiles of various inflammatory markers. The results showed that, compared to the T1 group, both the T2 and T3 groups had significantly higher rates of rehospitalization and death within one year, with this trend continuing to increase (*P* for trend <0.001). Figure 2 illustrates the rehospitalization rates within one year for the three inflammatory markers across the three groups, while Figure 3 shows the death rates within one year for these markers.

### 3.3 Relationship between various new inflammatory markers and re-hospitalization within one year

To investigate the relationship between different novel inflammatory markers and re-hospitalization in patients with HF within one year, we conducted a multivariate Cox regression analysis. The results revealed that as the levels of inflammatory markers increased, the risk of re-hospitalization also significantly increased. Specifically, in Model 1, for each one standard deviation (SD) increase in SIRI, SII, and NLR, the corresponding hazard ratios (HRs) were 1.240 [95% confidence interval (CI): 1.207–1.273], 1.456 (95% CI: 1.401–1.513), and 1.433 (95% CI: 1.374–1.495), respectively. These findings remained consistent in the fully adjusted Model 5.

**Figure 4:**
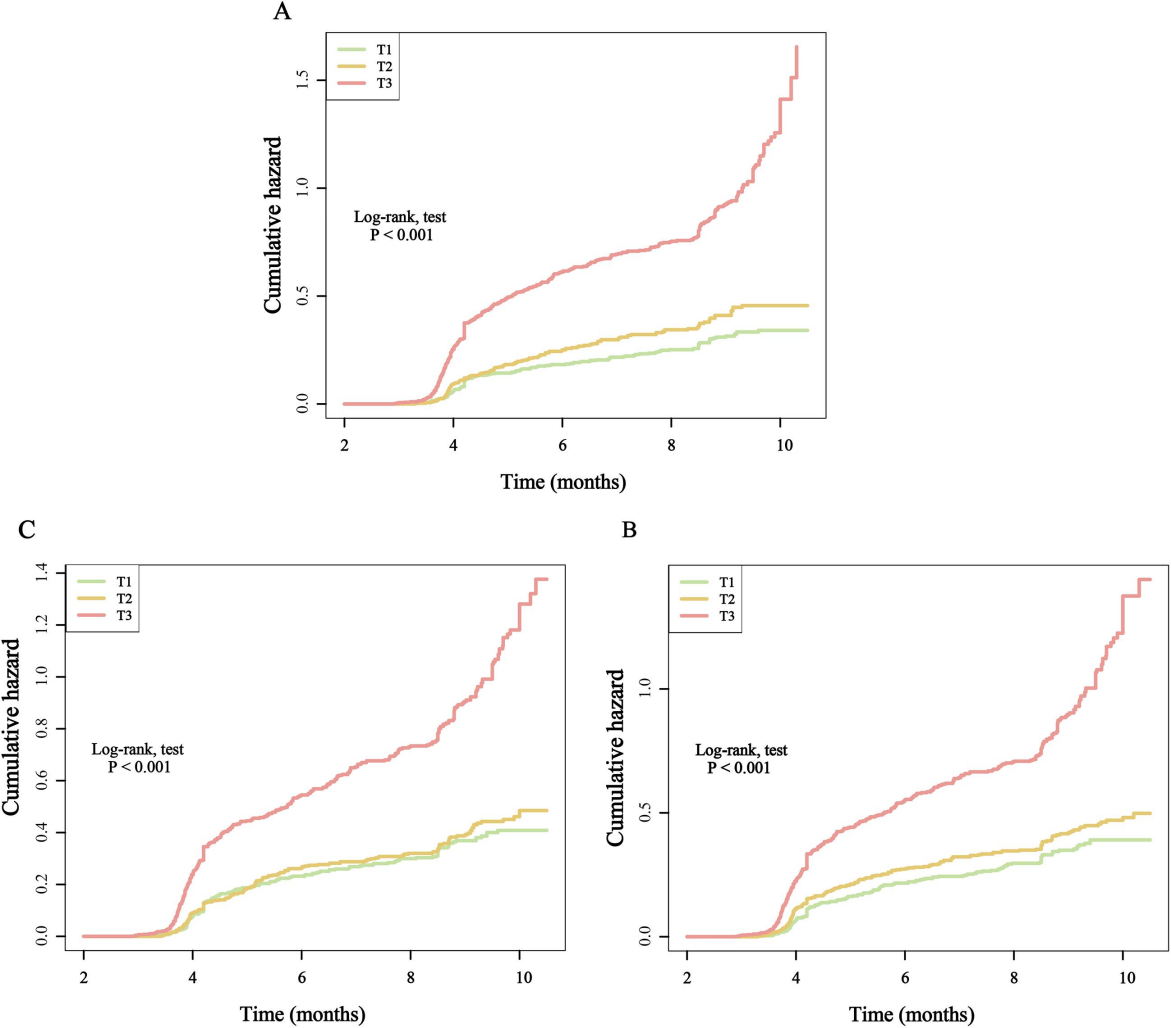
Kaplan–Meier cumulative risk curve of re-hospitalization within one year based on the tertiles of different inflammatory markers. **(A)**, SIRI; **(B)**, SII; **(C)**, NLR.

Furthermore, after categorizing the three inflammatory markers into T1, T2, and T3 groups, we found that compared to the T1 group, the re-hospitalization risk was significantly higher in both the T2 and T3 groups. The trend test was statistically significant, demonstrating a clear increasing trend (Table 2). To present these relationships more intuitively, we plotted the cumulative risk curves for the three groups. The results showed that, regardless of the inflammatory marker used, the re-hospitalization risk was significantly higher in the T3 group compared to the T1 and T2 groups, with log-rank test results being statistically significant (Figure 4). Finally, considering the gender differences, after stratification by gender, it is completely consistent with the overall results (Supplementary Table S1).

**Table 2:** Relationship between various novel inflammatory markers and re-hospitalization within one year in patients with heart failure.

Re-hospitalization	Model 1	Model 2	Model 3	Model 4
	HR (95% CI) P	HR (95% CI) P	HR (95% CI) P	HR (95% CI) P
SIRI				
SIRI (per 1SD increase)	1.240 [1.207, 1.273] <0.001	1.356 [1.312, 1.402] <0.001	1.357 [1.310, 1.406] <0.001	1.455 [1.403, 1.509] <0.001
Tertiles of SIRI				
Tertile 1	Reference	Reference	Reference	Reference
Tertile 2	1.263 [1.019, 1.566] 0.033	1.234 [0.995, 1.530] 0.055	1.230 [0.991, 1.526] 0.061	2.167 [1.729, 2.715] <0.001
Tertile 3	3.382 [2.827, 4.046] <0.001	3.380 [2.824, 4.046] <0.001	3.413 [2.851, 4.086] <0.001	6.211 [5.128, 7.524] <0.001
*P* for trend	<0.001	<0.001	<0.001	<0.001
SII				
SII (per 1SD increase)	1.456 [1.401, 1.513] <0.001	1.464 [1.407, 1.523] <0.001	1.491 [1.432, 1.552] <0.001	1.549 [1.489, 1.611] <0.001
Tertiles of SII				
Tertile 1	Reference	Reference	Reference	Reference
Tertile 2	1.243 [1.017, 1.519] 0.034	1.217 [0.995, 1.488] 0.056	1.236 [1.010, 1.513] 0.040	1.537 [1.253, 1.885] <0.001
Tertile 3	2.846 [2.382, 3.399] <0.001	2.740 [2.294, 3.274] <0.001	2.845 [2.379, 3.403] <0.001	3.975 [3.301, 4.787] <0.001
*P* for trend	<0.001	<0.001	<0.001	<0.001
NLR				
NLR (per 1SD increase)	1.433 [1.374, 1.495] <0.001	1.570 [1.496, 1.647] <0.001	1.575 [1.499, 1.655] <0.001	1.637 [1.562, 1.715] <0.001
Tertiles of NLR				
Tertile 1	Reference	Reference	Reference	Reference
Tertile 2	1.087 [0.889, 1.329] 0.415	1.068 [0.873, 1.307] 0.521	1.065 [0.870, 1.303] 0.542	1.334 [1.088, 1.636] 0.006
Tertile 3	2.671 [2.244, 3.179] <0.001	2.601 [2.184, 3.097] <0.001	2.619 [2.198, 3.121] <0.001	3.802 [3.167, 4.564] <0.001
*P* for trend	<0.001	<0.001	<0.001	<0.001

Model 1: no covariates were adjusted.

Model 2: age, sex, BMI, smoking status and drinking status were adjusted.

Model 3: Model 2 plus adjustment for SBP, DBP, ALT, AST, TC, TG, HDL.C, LDL.C, BNP, and FPG.

Model 4: Model 3 plus adjustment for DM, Dyslipidemia and CHD.

SIRI, Systemic Inflammation Response Index; SII, Systemic Immune-Inflammation Index; NLR, Neutrophil-to-Lymphocyte Ratio; HR, hazard ratio; CI, confidence interval.

Other abbreviations, see Table 1.

**Figure 5:**
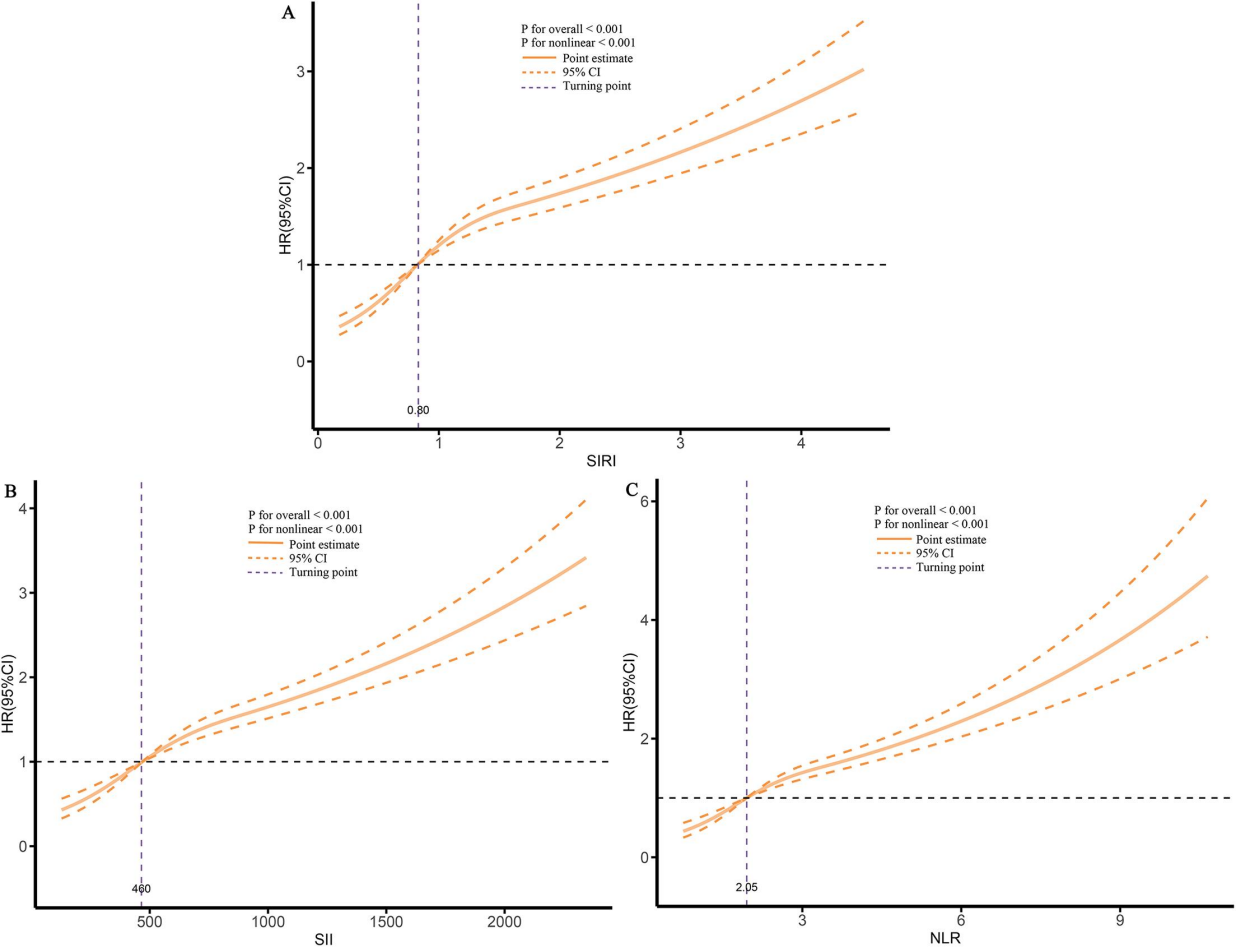
Dose-response association between different inflammatory markers and the risk of re-hospitalization. **(A)**, SIRI; **(B)**, SII; **(C)**, NLR.

Finally, using RCS analysis, we further examined the dose-response relationship between inflammatory markers and re-hospitalization risk. The results showed a significant increasing trend, indicating that higher levels of inflammatory markers are closely associated with an increased risk of future re-hospitalization (Figure 5).

### 3.4 Relationship between various new inflammatory markers and the risk of death within one year

We also analyzed the relationship between different novel inflammatory markers and the risk of death within one year in patients with HF. The results showed that, in the fully adjusted Model 5, for every 1 SD increase in SIRI, SII, and NLR, the corresponding risk of death increased by 63.8%, 70.1%, and 92.9%, respectively.

**Figure 6:**
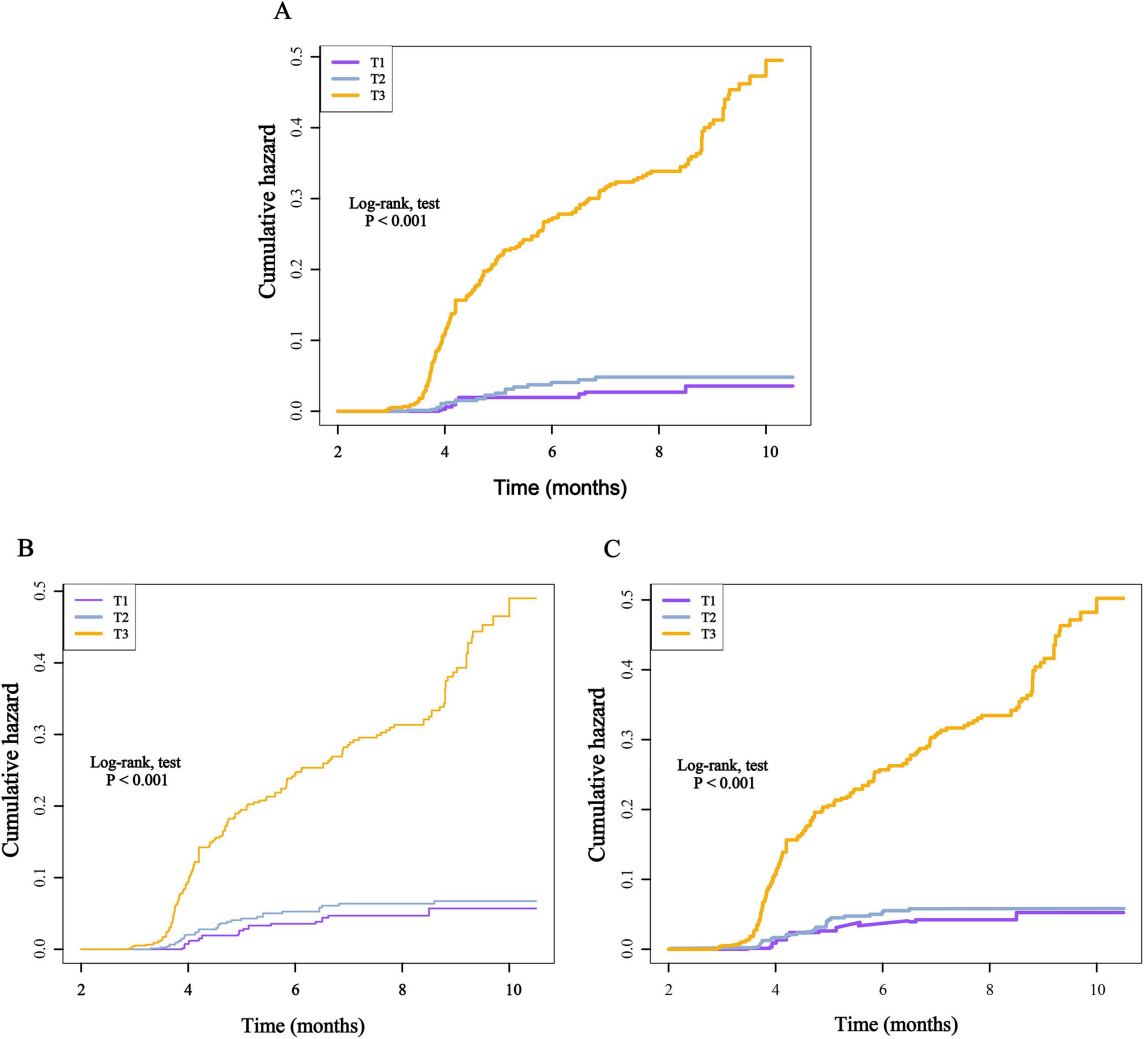
Kaplan–Meier cumulative risk curve of death within one year based on the tertiles of different inflammatory markers. **(A)**, SIRI; **(B)**, SII; **(C)**, NLR.

**Figure 7:**
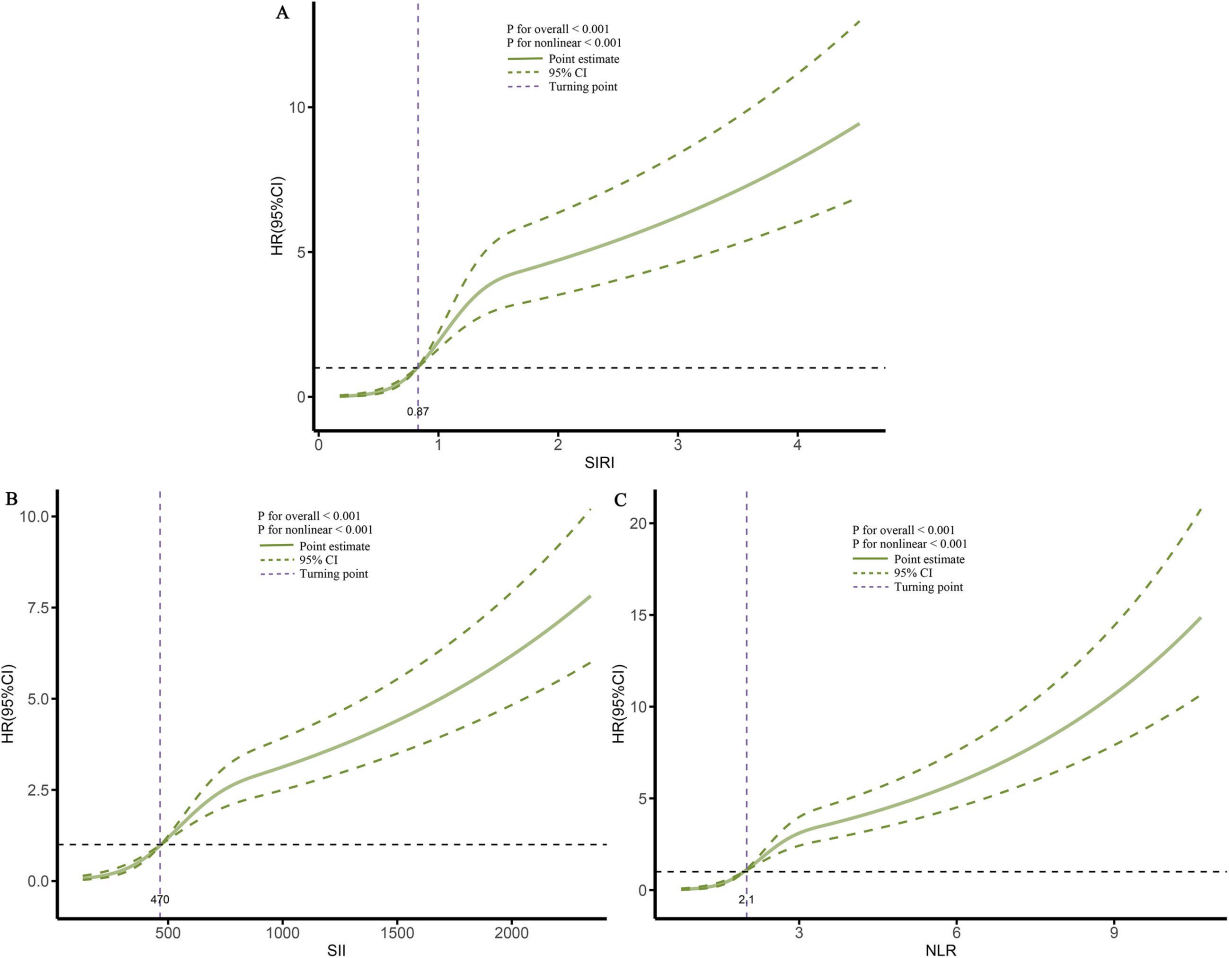
Dose-response association between different inflammatory markers and the risk of death. **(A)**, SIRI; **(B)**, SII; **(C)**, NLR.

When these markers were converted into categorical variables, the risk of death continued to show an increasing trend (Table 3). Additionally, the KM curve demonstrated that the risk of death in the T3 group was significantly higher than that in the T1 and T2 groups (Figure 6). Furthermore, the dose-response relationship based on RCS analysis confirmed these findings (Figure 7). Of course, even after stratification by gender, the results remained unchanged (Supplementary Table S2).

**Table 3:** Relationship between various novel inflammatory markers and the 1-year death risk of patients with heart failure.

Death	Model 1	Model 2	Model 3	Model 4
	HR (95% CI) P	HR (95% CI) P	HR (95% CI) P	HR (95% CI) P
SIRI				
SIRI (per 1SD increase)	1.334 [1.293, 1.375] <0.001	1.580 [1.503, 1.660] <0.001	1.612 [1.527, 1.702] <0.001	1.638 [1.548, 1.734] <0.001
Tertiles of SIRI				
Tertile 1	Reference	Reference	Reference	Reference
Tertile 2	1.378 [0.739, 2.571] 0.313	1.344 [0.720, 2.509] 0.353	1.361 [0.728, 2.544] 0.334	1.688 [0.890, 3.203] 0.109
Tertile 3	12.271 [7.578, 19.870] <0.001	12.449 [7.681, 20.177] <0.001	12.664 [7.810, 20.535] <0.001	16.179 [9.772, 26.784] <0.001
*P* for trend	<0.001	<0.001	<0.001	<0.001
SII				
SII (per 1SD increase)	1.604 [1.531, 1.680] <0.001	1.657 [1.577, 1.742] <0.001	1.685 [1.600, 1.775] <0.001	1.701 [1.613, 1.794] <0.001
Tertiles of SII				
Tertile 1	Reference	Reference	Reference	Reference
Tertile 2	1.337 [0.807, 2.214] 0.430	1.284 [0.775, 2.127] 0.455	1.290 [0.777, 2.140] 0.403	1.350 [0.810, 2.249] 0.396
Tertile 3	7.585 [5.028, 11.440] <0.001	7.228 [4.791, 10.906] <0.001	7.471 [4.941, 11.295] <0.001	7.974 [5.213, 12.197] <0.001
*P* for trend	<0.001	<0.001	<0.001	<0.001
NLR				
NLR (per 1SD increase)	1.625 [1.548, 1.705] <0.001	1.914 [1.787, 2.050] <0.001	1.925 [1.795, 2.064] <0.001	1.929 [1.799, 2.068] <0.001
Tertiles of NLR				
Tertile 1	Reference	Reference	Reference	Reference
Tertile 2	0.912 [0.521, 1.596] 0.746	0.880 [0.502, 1.542] 0.655	0.876 [0.500, 1.536] 0.645	0.935 [0.532, 1.647] 0.817
Tertile 3	8.287 [5.465, 12.568] <0.001	8.012 [5.278, 12.162] <0.001	8.069 [5.312, 12.257] <0.001	9.014 [5.854, 13.879] <0.001
*P* for trend	<0.001	<0.001	<0.001	<0.001

Model 1: no covariates were adjusted.

Model 2: age, sex, BMI, smoking status and drinking status were adjusted.

Model 3: Model 2 plus adjustment for SBP, DBP, ALT, AST, TC, TG, HDL.C, LDL.C, BNP, and FPG.

Model 4: Model 3 plus adjustment for DM, Dyslipidemia and CHD.

Other abbreviations, see Table 1.

SIRI, systemic inflammation response index; SII, systemic immune-inflammation index; NLR, neutrophil-to-lymphocyte ratio; HR, hazard ratio; CI, confidence interval.

In summary, these results reinforce the conclusion that higher levels of inflammatory markers are associated with an increased risk of adverse outcomes. This suggests that controlling inflammatory levels could potentially prevent and reduce adverse outcomes in patients with HF, thereby improving their quality of life and prognosis.

### 3.5 Comparative analysis of the predictive performance of various novel inflammatory markers for adverse outcomes

**Figure 8:**
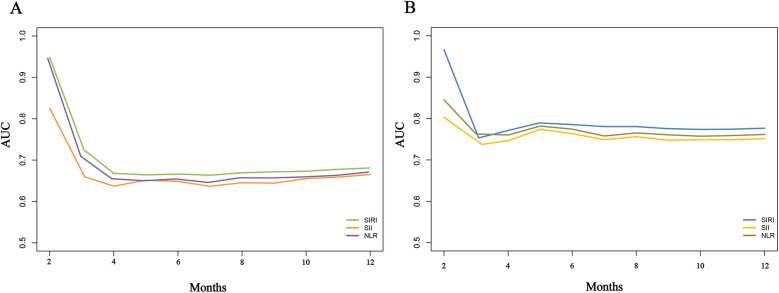
Time-dependent ROC curves for comparing the predictive value of different inflammatory markers for adverse outcomes in heart failure patients. **(A)**, Re-hospitalization; **(B)**, death.

To compare the predictive capabilities of different inflammatory markers for adverse outcomes, we first analyzed the area under the curve (AUC) of each indicator. The results showed that all three markers exhibited good predictive performance. Specifically, for predicting the risk of re-hospitalization within one year, the AUCs of SIRI, SII, and NLR were 0.747, 0.693, and 0.698, respectively, with SIRI demonstrating relatively better predictive ability (Table 4). For predicting death risk, the AUC of SIRI reached 0.866, which was significantly higher than that of the other inflammatory markers (Table 4). In the time-dependent ROC analysis, SIRI consistently outperformed the other indicators, showing superior predictive performance for both readmission and death risk (Figure 8).

**Table 4:** Comparative analysis of ROC curves for various novel inflammatory markers in predicting adverse outcomes within one year in patients with heart failure.

Inflammatory markers	AUC	95%CI low	95%CI up	Specificity	Sensitivity	Positive-pv	Negative-pv
Re-hospitalization							
SIRI	0.747	0.724	0.770	0.931	0.498	0.798	0.772
SII	0.693	0.670	0.717	0.799	0.513	0.583	0.750
NLR	0.698	0.674	0.721	0.759	0.564	0.562	0.761
Death							
SIRI	0.866	0.834	0.898	0.910	0.725	0.485	0.966
SII	0.797	0.763	0.831	0.709	0.765	0.261	0.957
NLR	0.826	0.793	0.858	0.872	0.664	0.379	0.957

Other abbreviations, see Table 1.

SIRI, systemic inflammation response index; SII, systemic immune-inflammation index; NLR, neutrophil-to-lymphocyte ratio; AUC, area under the curve; Positive-pv, positive predictive value; Negative-pv, negative predictive value.

Finally, based on the fully adjusted Model 4, we conducted a C-statistic analysis and added SIRI, SII, and NLR to the model sequentially. The C-statistics for one-year readmission outcomes were 0.751, 0.737, and 0.740, respectively, while the C-statistics for one-year death outcomes were 0.805, 0.791, and 0.801. These results indicated that while all markers demonstrated good predictive capabilities, SIRI still showed the best performance (Table 5).

**Table 5:** Comparative analysis of various novel inflammatory markers in predicting adverse outcomes within one year for patients with heart failure.

Inflammatory markers	C-index
Re-hospitalization	
Model 4	0.686
+SIRI	0.751
+SII	0.737
+NLR	0.740
Death	
Model 4	0.664
+SIRI	0.805
+SII	0.791
+NLR	0.801

Other abbreviations, see Table 1.

SIRI, systemic inflammation response index; SII, systemic immune-inflammation index; NLR, neutrophil-to-lymphocyte ratio.

In conclusion, these comparative analyses further suggest that SIRI, as a systemic inflammatory response index, holds significant clinical value as an indicator for assessing future adverse outcomes in patients with HF.

## 4 Discussion

In this longitudinal cohort study, we investigated the relationship between three novel inflammatory markers (SIRI, SII, NLR) and adverse outcomes in HF patients within one year. The Cox regression analysis showed that elevated levels of these inflammatory markers were significantly associated with an increased risk of adverse outcomes. Even after fully adjusting for various confounding factors, the results remained consistent. Additionally, the dose-response relationship, established using RCS, confirmed this trend. The KM survival curve demonstrated that the T3 group, with higher inflammatory levels, had a significantly greater risk of adverse outcomes compared to the T1 and T2 groups, which had lower levels of inflammation. Furthermore, we compared the predictive performance of the three inflammatory markers using single ROC curves, time-dependent ROC curves, and C-statistics. The results showed that the SIRI exhibited the best predictive ability, further confirming its reliability as a predictor of adverse outcomes in HF patients. Based on these results, positioning SIRI as a useful biomarker for assessing the risk of adverse outcomes in HF patients holds significant clinical value. Moreover, early control of inflammatory levels may help prevent or delay the onset of adverse outcomes, offering potential intervention strategies for clinical practice.

HF is a condition caused by the dysfunction of the heart's pumping ability, preventing it from supplying the body with adequate blood, which leads to a range of clinical symptoms (1). Due to its high prevalence and mortality rate, HF has become a major public health concern. Global epidemiological studies indicate that the incidence of HF is rising annually, particularly in the context of an aging population, resulting in an increasing disease burden (3, 19, 20). According to the World Health Organization (WHO), HF holds a significant position among all CVD, with its prevalence reaching 20%–30% in the elderly population. This figure continues to rise, especially in underdeveloped regions and developing countries (21, 22). Furthermore, HF patients often have multiple comorbidities, including hypertension, diabetes, chronic obstructive pulmonary disease, and chronic kidney disease. These comorbidities not only increase the complexity of the disease but also significantly affect treatment outcomes (19, 23–25). Given the high risk and complexity associated with HF, identifying effective predictive markers and implementing early preventive measures are crucial to delaying or preventing adverse outcomes.

Inflammation, as a protective response of the body to injury, infection, or stimulation, plays a crucial role in the development of various diseases. Studies have shown that the inflammatory response can damage vascular endothelial cells, increasing the risk of cardiovascular and cerebrovascular diseases (6, 26). Chronic inflammation, in particular, can stimulate the immune and humoral regulatory systems, leading to immune imbalance or overactivation, which increases the risk of autoimmune diseases (27–29). Additionally, inflammation is commonly observed across multiple organ systems, such as in liver cirrhosis, pulmonary fibrosis, and myocardial fibrosis, all of which are closely associated with inflammation (6, 9, 29).

Given the critical role of inflammation in these diseases, novel inflammatory markers derived from whole blood cells—such as SIRI, SII, PLR, NLR, and MLR—have gained widespread attention. These markers are not only easy to calculate and obtain but also demonstrate superior predictive performance (9, 12, 13, 30). For example, a large-scale study by Shen et al. highlighted the significant role of SIRI, SII, and NLR in diseases such as hypertension and fatty liver, promoting liver degeneration and even cirrhosis (9). Similarly, inflammation plays an essential role in bone loss and osteoporosis. A study from Xinjiang, China, confirmed that elevated SIRI levels are closely linked to the development of osteoporosis, suggesting that controlling inflammation could help prevent bone loss and damage (12). Inflammation is also crucial in CVD. A cohort study demonstrated that SIRI outperforms other indicators in predicting the risk of stroke in patients with hypertension, showing excellent predictive ability (14). Additionally, in sepsis research, SIRI was found to be superior to traditional indicators, such as white blood cells, procalcitonin, and CRP, in predicting patient mortality (31). Our findings are consistent with these results: as the levels of inflammatory markers increase, the risk of adverse outcomes in HF patients rises significantly. Among the three inflammatory markers, SIRI demonstrates particularly strong predictive ability. In summary, these studies collectively demonstrate that new inflammatory markers offer a more comprehensive reflection of the body's inflammatory state compared to traditional indicators. This discovery has significant clinical implications: it provides a valuable tool for monitoring inflammation levels in patients, enabling early prevention and intervention in disease progression, and reducing the occurrence of adverse outcomes.

Inflammation plays a complex and diverse role in the onset and progression of HF, with its mechanism involving multiple interacting aspects. First, inflammation promotes myocardial injury and remodeling: the inflammatory response induces cardiomyocyte apoptosis and fibrosis. This process accelerates ventricular remodeling, impairs the heart's pumping capacity, and thus drives disease progression (32–34). Second, elevated inflammatory mediators cause myocardial cell injury and necrosis (34, 35). In HF, multiple inflammatory factors—such as tumor necrosis factor-alpha (TNF-α), interleukin-6 (IL-6), IL-1β, and CRP—are increased. These factors participate in the inflammatory process and also affect myocardial cell function and survival, contributing to enhanced cell death (35–38). Moreover, the inflammatory response can worsen coronary microcirculation disorders through oxidative stress and endothelial dysfunction, aggravating myocardial ischemia and energy metabolism disturbances (39, 40). In acute decompensated HF, higher levels of inflammatory markers (e.g., CRP, galectin-3) correlate with clinical deterioration, possibly by activating systemic immune responses and promoting fluid retention and insufficient organ perfusion (39–43). Studies indicate that persistently elevated inflammatory factors independently predict HF readmission and all-cause mortality and may relate to the progression of multiorgan failure and cachexia (44, 45). Additionally, inflammation activates the immune system: HF patients often exhibit systemic inflammatory activation, which can affect other organs (such as the kidneys and liver) and lead to multisystem dysfunction (45–47). In advanced HF, the role of inflammation becomes even more crucial. Inflammation can amplify cardiomyocyte apoptosis and necrosis, promote fibrosis, and reduce cardiac elasticity, thereby exacerbating endothelial dysfunction and hemodynamic instability (48–51). Collectively, these interconnected mechanisms contribute to adverse outcomes and increased risk of death.

The advantage of this study is that it is the first to perform a comprehensive statistical analysis based on a large sample and rich clinical data, enabling a deep exploration of the relationship between different novel inflammatory markers and the risk of adverse outcomes in patients with HF. In the comparative analysis, we identified the most effective clinical predictive indicators, which hold significant implications for future clinical monitoring of inflammatory conditions in HF patients as well as for early prevention and intervention of adverse outcomes. Of course, despite these advantages, the study has some limitations. First, we only collected baseline blood test information, and laboratory values may fluctuate due to various factors; future studies should collect multiple blood test data over time to validate and refine these findings. Second, the study population was drawn entirely from southwest China, and the gender distribution was skewed toward females; caution is needed when generalizing the results to other regions or to different gender compositions. Finally, we did not collect data on the use of anti-inflammatory drugs and some novel therapies for HF, as this could affect the study results; future research should address this gap.

## 5 Conclusion

This study is the first to systematically clarify the close correlation between multiple novel inflammatory markers and adverse outcomes in HF patients within one year. The results show that increases in inflammatory marker levels are significantly associated with a higher risk of adverse outcomes. Moreover, comparative analysis shows that SIRI has the best predictive performance in this study's data analysis. This finding highlights the potential value of SIRI for clinical monitoring of HF patients and for the early identification of high-risk individuals. At the same time, the findings suggest that actively managing inflammation in clinical practice may help prevent or slow the progression of adverse outcomes and thereby improve patients' prognosis.

## Data Availability

The raw data supporting the conclusions of this article will be made available by the authors, without undue reservation.
