# Supplementary material for: Elevated novel inflammatory markers in heart failure patients are associated with increased risk of adverse outcomes within one year: insights from a longitudinal study
Source: Front Cardiovasc Med. 2025 Sep 25;12:1683273. doi: 10.3389/fcvm.2025.1683273 (PMC12507900; doi:10.3389/fcvm.2025.1683273)
Supplement: Supplementary file 1 [file Datasheet1.pdf]

Supplementary Material

1 Supplemental material and methods

Baseline examination

Anthropometric measurements were taken by trained nurses. Data for height and weight were 3 acquired following a protocol standardized to an accuracy of 0.1 kg and 0.1 cm, respectively. Current smokers were defined as having smoked 100 cigarettes in their lifetime and currently smoking. Alcohol consumption was evaluated with questions regarding the types of alcoholic beverages, the frequency of alcohol consumption per week, and the usual amount consumed per occasion. Subjects who reported alcohol consumption >140 g/week for men and >70 g/week for women were deemed to have excessive alcohol consumption (1).

Definitions

Criteria for hypertension included self-reported hypertension, current use of anti-hypertensive medication, or systolic blood pressure (SBP) ≥ 140 mmHg and/or diastolic blood pressure (DBP) ≥ 90 mmHg recorded for at least three consecutive readings. diabetes was defined as fasting serum glucose ≥7.0 mmol/L, the 2-h serum glucose of the oral glucose tolerance test ≥11.1 mmol/L, or the current use of hypoglycaemic medication or insulin. coronary heart disease (CHD) was defined as a fatal or nonfatal myocardial infarction, unstable angina, and coronary revascularization. Hyperlipidemia is defined as an abnormal elevation of lipid levels in the bloodstream, primarily characterized by increased concentrations of total cholesterol (TC), low-density lipoprotein cholesterol (LDL-C), and triglycerides (TG), or a reduction in high-density lipoprotein cholesterol (HDL-C). According to international guidelines, hyperlipidemia is diagnosed when TC levels exceed 6.2 mmol/L (240 mg/dL), LDL-C levels are ⩾ 4.1 mmol/L (160 mg/dL), TG levels are ⩾ 2.3 mmol/L (200 mg/dL), or HDL-C levels fall below 1.0 mmol/L (40 mg/dL) in men and 1.3 mmol/L (50 mg/dL) in women. Cancer was based on self-report of

physician diagnosis of cancer or malignancy and/or use of anticancer medications. The body mass index (BMI) was calculated as per the formula: Weight (kg)/Height^2 (m).

Details of the statistical analyses.

Variables of baseline characteristics are shown as n (%) if categorical, mean (SD) if normally distributed, and median (interquartile range) if nonnormally distributed. To compare the characteristics among different inflammatory markers groups, the chi-square test was performed for categorical variables, and one-way analysis of variance, or the Kruskal-Wallis test, was performed for continuous variables with normal and skewed distributions.

The association between inflammatory markers and adverse outcomes within one year was tested with multivariable Cox regression models. This study set four different models (Model 1: unadjusted; Model 2: age, sex, BMI, smoking status and drinking status were adjusted; Model 3:Model 2 plus adjustment for SBP, DBP, ALT, AST, TC, TG, HDL.C, LDL.C, and FPG. Model 4: Model 3 plus adjustment for DM,Dyslipidemia and CHD.) to adjust for covariates considering that the condition of over-adjustment might exist when a large number of factors are adjusted for simultaneously. Tests for trend were conducted, assigning the median value within each tertile to the corresponding tertile. In addition, the restricted inverse square spline (four nodes at the 5th, 35th, 65th, and 95th percentiles of the inflammatory markers distribution) was used to evaluate the nonlinear relationships. Finally, receiver operating characteristic (ROC) curves, time-dependent ROC curves, and C statistics were applied to compare the predictive performance of these markers.

All analyses were done using R (4.2.2). All P-values were two-sided, and P-values of <0.05 denoted statistical significance.

2 Supplementary Tables

Table S1. Relationship between various novel inflammatory markers and re-hospitalization within one year in patients with heart failure after stratification by gender

Re-hospitalization	Model 1	Model 2	Model 3	Model 4
	HR (95% CI) P	HR (95% CI) P	HR (95% CI) P	HR (95% CI) P
**SIRI**				
**Female**				
SIRI (per 1SD increase)	1.782 [1.693, 1.876] <0.001	1.762 [1.669, 1.861] <0.001	1.749 [1.653, 1.850] <0.001	1.966 [1.857, 2.082] <0.001
**Male**				
SIRI (per 1SD increase)	1.175 [1.120, 1.233] <0.001	1.207 [1.144, 1.274] <0.001	1.225 [1.146, 1.309] <0.001	1.296 [1.208, 1.389] <0.001
**SII**				
**Female**				
SII (per 1SD increase)	1.445 [1.380, 1.513] <0.001	1.451 [1.384, 1.522] <0.001	1.483 [1.412, 1.557] <0.001	1.586 [1.512, 1.664] <0.001
**Male**				
SII (per 1SD increase)	1.585 [1.435, 1.751] <0.001	1.565 [1.416, 1.729] <0.001	1.552 [1.405, 1.714] <0.001	1.577 [1.422, 1.749] <0.001
**NLR**				
**Female**				
NLR (per 1SD increase)	1.856 [1.721, 2.001] <0.001	1.908 [1.761, 2.067] <0.001	1.924 [1.772, 2.088] <0.001	2.169 [2.000, 2.353] <0.001

**Male**				
NLR (per 1SD increase)	1.374 [1.281, 1.473] <0.001	1.423 [1.316, 1.538] <0.001	1.438 [1.319, 1.569] <0.001	1.507 [1.380, 1.645] <0.001

Model 1: no covariates were adjusted.

Model 2: age, BMI, smoking status and drinking status were adjusted.

Model 3: Model 2 plus adjustment for SBP, DBP, ALT, AST, TC, TG, HDL.C, LDL.C, BNP, and FPG.

Model 4: Model 3 plus adjustment for DM,Dyslipidemia and CHD.

Abbreviations: SIRI, Systemic Inflammation Response Index; SII, Systemic Immune-Inflammation Index; NLR, Neutrophil-to-Lymphocyte Ratio; HR, hazard ratio; CI, confidence interval

Other abbreviations, see Table 1.

Table S2. Relationship between various novel inflammatory markers and the 1-year death risk of patients with heart failure after stratification by gender

Death	Model 1	Model 2	Model 3	Model 4
	HR (95% CI) P	HR (95% CI) P	HR (95% CI) P	HR (95% CI) P
**SIRI**				
**Female**				
SIRI (per 1SD increase)	2.242 [2.068, 2.431] <0.001	2.280 [2.105, 2.469] <0.001	2.364 [2.165, 2.582] <0.001	2.454 [2.235, 2.694] <0.001
**Male**				
SIRI (per 1SD increase)	1.244 [1.169, 1.323] <0.001	1.326 [1.224, 1.436] <0.001	1.366 [1.212, 1.540] <0.001	1.427 [1.261, 1.614] <0.001
**SII**				
**Female**				
SII (per 1SD increase)	1.603 [1.520, 1.690] <0.001	1.680 [1.582, 1.784] <0.001	1.719 [1.613, 1.833] <0.001	1.730 [1.618, 1.851] <0.001
**Male**				
SII (per 1SD increase)	1.908 [1.539, 2.366] <0.001	1.921 [1.574, 2.345] <0.001	1.821 [1.550, 2.139] <0.001	1.862 [1.586, 2.186] <0.001
**NLR**				
**Female**				
NLR (per 1SD increase)	2.457 [2.257, 2.674] <0.001	2.733 [2.481, 3.009] <0.001	2.762 [2.499, 3.054] <0.001	2.827 [2.538, 3.150] <0.001
**Male**				

NLR (per 1SD increase)	1.524 [1.398, 1.661] <0.001	1.690 [1.501, 1.902] <0.001	1.705 [1.495, 1.945] <0.001	1.777 [1.552, 2.034] <0.001

Model 1: no covariates were adjusted.

Model 2: age, BMI, smoking status and drinking status were adjusted.

Model 3: Model 2 plus adjustment for SBP, DBP, ALT, AST, TC, TG, HDL.C, LDL.C, BNP, and FPG.

Model 4: Model 3 plus adjustment for DM,Dyslipidemia and CHD.

Abbreviations: SIRI, Systemic Inflammation Response Index; SII, Systemic Immune-Inflammation Index; NLR, Neutrophil-to-Lymphocyte Ratio; HR, hazard ratio; CI, confidence interval

Other abbreviations, see Table 1.

References

1. Farrell GC, Chitturi S, Lau GK, Sollano JD. Guidelines for the assessment and management of non-alcoholic fatty liver disease in the Asia-Pacific region: executive summary. Journal of gastroenterology and hepatology. 2007;22(6):775-7. Epub 2007/06/15. doi: 10.1111/j.1440-1746.2007.05002.x. PubMed PMID: 17565629.
